# Population-based incidences of non-fatal injuries - results of the German-wide telephone survey 2004

**DOI:** 10.1186/1471-2458-13-376

**Published:** 2013-04-22

**Authors:** Anke-Christine Sass, Andreas Stang

**Affiliations:** 1 Department of Epidemiology and Health Monitoring, Robert Koch Institute, General-Pape-Str. 62, Berlin, 12101, Germany; 2 Institute of Clinical Epidemiology, Medical Faculty, Martin-Luther-University of Halle-Wittenberg, Magdeburger Str. 8, Halle, 06097, Germany

**Keywords:** Epidemiology, Germany, Injuries, Accidents, Traffic accidents, Domestic accidents, Leisure-time accidents, Work-related accidents, Incidence, Health survey

## Abstract

**Background:**

To plan preventive measures against accident-related injuries, it is important to have detailed epidemiological data on this topic. The aim of this report was to present population-based incidence estimates of injuries due to non-fatal accidents in relation to age, gender and educational level.

**Methods:**

We performed a cross-sectional telephone survey from 2003 to 2004 of the resident adult population of Germany, which included 7,341 subjects (response rate: 32.6 to 39.4%). The interview included 13 questions about injuries caused by accidents that happened in the 12 months preceding the interview. We estimated one-year cumulative incidences of injuries by gender, age and educational level.

**Results:**

Overall, 10.3% of the subjects reported an unintentional injury requiring medical treatment in the previous 12 months. The age-standardised incidence of injuries was higher among men than women (men: 11.3%, women: 8.9%). Generally, accidents at home were the most frequently reported (27.4%). Men and women aged 18 to 29 years suffered accident-related injuries (and also repeated injuries) the most often during the preceding 12 months.

Although the overall incidence of injuries caused by accidents did not differ by educational level, the incidences of accidents at different places differed by educational level. The incidence of work-related injuries was higher among people with a low educational level.

**Conclusions:**

Our age- and gender-specific results provide detailed insight into specific patterns of accident-related injuries in Germany. Young men are especially at high risk of injuries. This information is valuable because a nationwide comprehensive recording of injuries caused by accidents does not exist. The data highlight the target groups for injury prevention measures.

## Background

One person dies every two minutes from an injury in Europe (27 Member States) and about 256,000 people die from accidents or violent attacks per year. Injuries are the fourth most common cause of death in the European Union [1]. In 2010, 20,243 fatal injuries (2.4% of all 858,768 deaths) occurred in Germany alone [2]. It has been estimated that about 8.25 million people were injured in 2010 according to a report by the Federal Institute for Occupational Safety and Health (Bundesanstalt für Arbeitsschutz und Arbeitsmedizin) [3]. These figures emphasise the public health importance of injuries in relation to potential years of life lost, physical impairment, long-term disability and societal costs due to sick leave and lost years of gainful employment. The estimated costs of accidents related to injury, poisoning and other causes (International Classification of Diseases and Related Health Problems [4], ICD-10: S00-T98) were about 12.6 billion Euros (about 4.9% of the total disease cost) in 2008, according to the Federal Bureau of Statistics in Germany [5].

Unintentional injuries are responsible for about three-quarters of all injury deaths in the European Union and for about two-thirds of the injury deaths in Germany [1,2].

To plan targeted preventive measures against accidents in Germany, it is important to have detailed epidemiological data on injuries caused by accidents in relation to gender, age and circumstances. In Germany, police documentation provides statistics on registered road traffic accidents [6]. Industrial and school accidents – those that fall under the legal responsibility of accident insurance – are also subject to registration [7]. However, injuries due to domestic and leisure-time accidents are not routinely registered. These places of accidents are of particular interest as two-thirds (65.0%) of those injured in 2010 experienced accidents at home or during leisure activities [3]. An additional source of information is obtained from the statistics related to the cause of death [2]. However, this source presents very limited data on overall injury occurrence as only a fraction of injuries are lethal. Some statistics on the accidents mentioned above overlap substantially, others are restricted to only certain population groups, while most are not methodologically comparable. Injuries due to traffic accidents, for example, are only registered when police are present at the scene. If the injured person was en route to work, this incident is also recorded by the statutory accident insurance carriers as an accident at work (work-related road accident).

The aim of this report was to present population-based incidence estimates of injuries caused by accidents in relation to age, gender and educational level (as proxy for socioeconomic status), based on a German-wide telephone survey conducted by the Robert Koch Institute in 2004.

## Methods

The telephone health survey is an annual cross-sectional study of the resident population of Germany. It provides information on the prevalence of chronic diseases and their risk factors, individual perceptions on health, health behaviour and health care utilization [8]. Every year, there are certain priority themes, e.g., accident-related injuries in 2004. For this survey, telephone recruitment took place between September 29, 2003, and March 6, 2004. The study was designed to represent the German-speaking adult population living in private households that could be contacted by landline telephones. The survey sample was based on randomly generated telephone numbers, according to the Gabler-Haeder design [9,10]. The design also included households that were not listed in the public phone directory. The random selection of people within households followed the next birthday method, in which the person whose birthday was coming soon was chosen for interview.

The interview in the telephone health survey of 2004 included 13 questions about accident-related injuries that happened in the 12 months preceding the interview. The accident-related questionnaire was introduced by a brief explanation: “The next few questions are about unintentional injuries due to accidents. Injuries as a result of assaults or self-harming are not included.” The first question was: “Did you have an accident-related injury or intoxication in the 12 months before this interview that was treated by a physician?”. Further questions were asked concerning the location, type of body part injured and consequences of the accidents. The respondents could select from four locations: at home, at another place during leisure-time, at workplace and on public roads and places. In this report we call this category "road traffic accidents" (it includes self-inflicted injuries of pedestrians, e.g. falls).

Injuries by force or self-injuries were not recorded. If more than one accident-related injury occurred during the 12 months before the interview, that number was recorded. Places of accidents were cumulatively documented. As an example, for a subject reporting two unintentional injuries at the workplace and one injury at home, two (workplace and home) locations were documented although three separate accidents had occurred. Therefore, the assignment of places of occurrence became ambiguous if the number of accidents did not equal the number of places of accidents. Accidents that reportedly occurred at unclearly defined places were assigned to the category “unknown places”. For subjects reporting more than one accident-related injury but only one place of accident, the place of the accidents was then clear.

**Figure 1:**
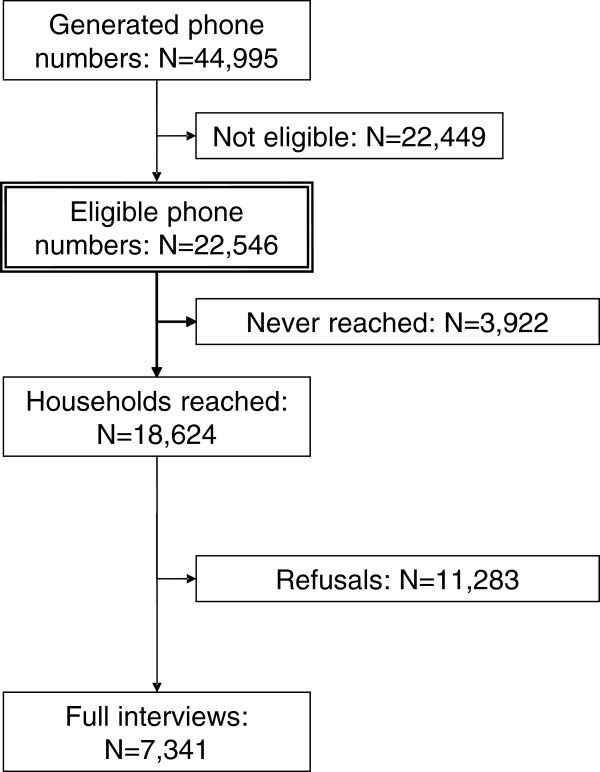
**Recruitment results of the German National Telephone Survey 2004.** Not eligible: the generated phone numbers did not exist, phone numbers were non-private household numbers, and phone numbers were fax or modem numbers.

Educational level was measured by the highest school degree and highest post-school training. We used an international classification of educational level, the CASMIN classification (Comparative Analysis of Social Mobility in Industrial Nations; low, middle and high educational level), to process information on educational level [11]. Figure 1 presents the recruitment results. Overall, 44,995 phone numbers were randomly generated and 7,341 people completed full interviews (22,449 were not eligible phone numbers, i.e., the phone numbers did not exist/were non-private household numbers, fax or modem numbers). Since 3,825 potentially eligible households could not be reached, the response rate was somewhat uncertain. Assuming that all the households that had not been reached were eligible, the response rate would have been 32.6%. However, if all the households that had not been reached were not eligible, the response proportion would have been 39.4%. Thus, the response rate was somewhere between 32.6% and 39.4%.

All observations were weighted by a specific weighting factor accounting for the two-stage sampling design [8] to obtain representative estimates of the population in Germany. We estimated one-year cumulative incidences (injuries per 100 individuals) of unintentional injuries in relation to gender, age, educational level and places of accidents. For overall analysis, we calculated a cumulative incidence that accounted for only one injury in the 12-month period (“any”) and a cumulative incidence that also accounted for the number of injuries during the 12-month period (“all”). For work-related injuries, we restricted the denominator to the working population aged 18–69 years. We estimated the gender ratio (men/women) of the age-standardised cumulative incidences and the corresponding 95% confidence intervals according to the methods described by Boyle and Parkin [12]. We calculated age-specific and age-standardised cumulative incidences by gender. We used the official German population figures from December 31, 2003, for age standardisation as provided by the Federal Bureau of Statistics [13].

To estimate potential selection effects due to non-response, we compared the distribution of school degrees of our survey with the nationwide German census data from 2004 (Microcensus), a virtually unbiased sample of the German nation [14]. As age-specific distributions of school degrees of the census data were separated by age groups (15–19, 20–24, …, 60–64, 65+ years), we compared gender-specific age-standardised prevalence rates of school degrees for the age range of 20–64 years. The proportion of survey subjects with university entrance qualifications was considerably higher than that in the census. For example, the prevalence of the highest school degree (university entrance qualification) was 40.3% and 33.9% among men and women in the telephone survey. However, these rates were 28.8% and 25.4%, respectively, in the census data. Similarly, 26.0% and 23.9% of the men and women of the survey, respectively, had the lowest school degree, whereas 38.5% and 35.3%, respectively, had this school degree in the census data.

In a sensitivity analysis, we used gender- and age-specific weights of school degrees derived from the census to standardise the survey incidence estimates (age 20–64 years), with the gender- and age-specific school degree distribution of the census correcting for any potential non-response bias. All analyses were performed with SAS, version 9.1 [15].

Ethics approval was obtained from the ethics committee of the Berlin Medical Association (Eth-423/04).

## Results

Overall, 757 out of 7,341 participants (10.3%) reported a total of 909 injuries caused by accidents in the previous 12 months that required medical attention. Of these, 12.9% had been injured in more than one accident during this period and 16.4% had been treated in hospital (inpatient at least one night). With increasing age, there was a pronounced increase in hospitalisation (70–79 years: 39.0%).

Accidents at home were the most frequently reported accidents (27.4%). Overall, 200 injuries at the workplace were reported among the 4,783 working people (Table 1).

**Table 1:** Characteristics of the study participants

	**Number of study subjects**	**Employed subjects**	**Total number of accident-related injuries**	**Number of injuries by place of accident**					
				**Road traffic accident**	**Home accident**	**Leisure accident**	Accident at work*	**Other places**	**Unknown places**
**Age**									
18-29	1415	997	269	61	51	80	50	7	20
30-39	1728	1375	232	53	51	58	59	1	10
40-49	1699	1419	191	24	66	34	59	0	8
50-59	1040	779	101	21	31	16	29	0	4
60-69	930	194	70	21	34	8	2	0	5
70-79	415	17	33	14	12	5	1	0	1
80+	110	2	13	8	4	0	0	0	1
Total	7337	4783	909	202	249	201	200	8	49
**Educational level**									
Low	1951	1040	213	47	58	30	63	1	14
Middle	3647	2486	496	109	143	105	106	6	27
High	1552	1171	159	37	40	48	28	0	6
Unknown	187	86	41	9	8	18	3	1	2

Overall, 4 out of 7,341 were excluded due to missing data on injuries; all absolute numbers are unweighted. Employed: full-time or part-time; educational status based on the CASMIN educational classification [11].

909 accident-related injuries were reported among the 757 subjects.

*187 out of 200 reported work-related accidents occurred among employed subjects of any age.

Generally, the overall age-standardised incidence of injuries was higher among men than women (men: 11.3%, women: 8.9%). Among men, injuries due to work-related accidents were the most frequent. Among women, however, unintentional injuries at home had the highest incidence. Thus, gender was associated with the place of accident. Men had a 1.9- and 2.7-fold higher incidence of injuries due to leisure- and work-related accidents, respectively, than women. Only the age-standardised incidence of injuries caused by traffic accidents was higher among women (3.2%) than men (2.4%). The analysis of the type of traffic accidents revealed that men most often experienced accidents in cars, whereas women most often experienced accidents as pedestrians. The incidence of injuries due to pedestrian traffic accidents among women was about twice that of men (Table 2).

**Table 2:** Crude (CR) and age-standardised incidence rates (ASR) of unintentional injuries by gender and place of accident

	**Men**				**Women**				**Gender ratio (Men : Women)**	
	**N**	**CR**	**ASR**	**SE**	**N**	**CR**	**ASR**	**SE**	**Ratio**	**95%CI**
**Any injury** due to non-fatal accidents (total)	408	11.4	11.3	0.6	349	8.9	8.9	0.5	1.3	1.1-1.5
**All injuries** due to non-fatal accidents (total)	510	14.2	13.9	0.6	399	10.1	10.1	0.5	1.4	1.2-1.6
**Home** accident-related injuries	110	3.1	3.4	0.4	139	3.5	3.5	0.3	1.0	0.7-1.3
**Leisure** accident-related injuries	130	3.5	3.3	0.3	71	1.7	1.7	0.2	1.9	1.5-2.7
**Workplace**-related injuries*	135	5.6	5.1	05	51	2.3	1.9	0.3	2.7	1.9-3.7
**Road traffic** accident-related injuries	87	2.5	2.4	0.26	115	3.3	3.2	0.3	0.8	0.6-1.0
Bicycle	21	0.6	0.6	0.15	23	0.7	0.6	0.1	1.0	0.5-2.2
Pedestrian	27	0.8	0.8	0.15	47	1.5	1.5	0.2	0.5	0.3-0.8
Driver or passenger in another vehicle	37	1.1	1.0	0.16	42	1.0	1.0	0.2	1.0	0.7-1.6

N: unweighted number of injuries due to non-fatal accidents; CR: crude rate per 100 person years; ASR: age-standardised rate: weighted age-standardised incidence rate per 100 person years; SE: standard errors of the age-standardised incidence rates calculated using the binomial distribution [12].

Gender ratios: ratios of male to female age-standardised incidence rates.

*Age range restricted to people aged 18–69 years because only 19 people aged 70+ years were still employed.

**Figure 2:**
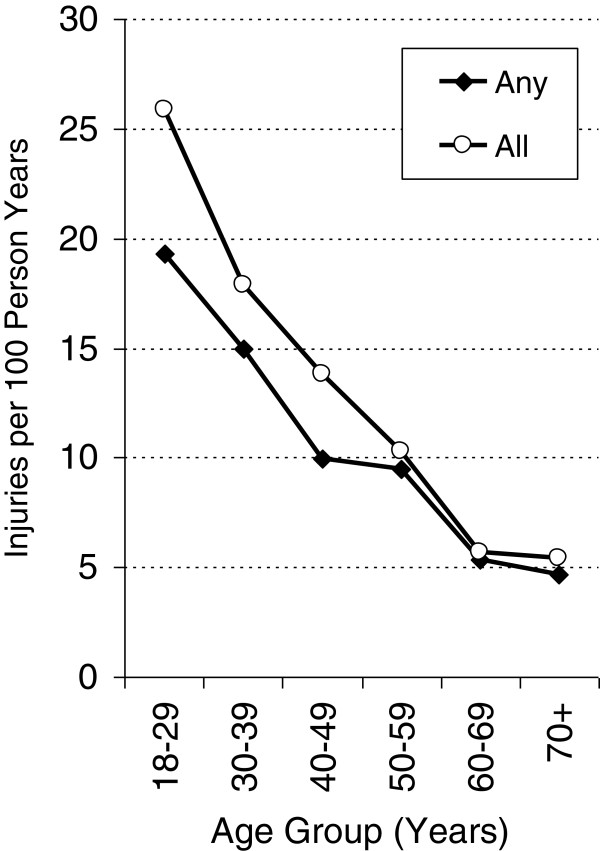
**Age-specific injury rates (per 100 person years) in men.** Any: incidence of any reported accidents during the previous 12 months; All: all reported accidents per 100 person years.

**Figure 3:**
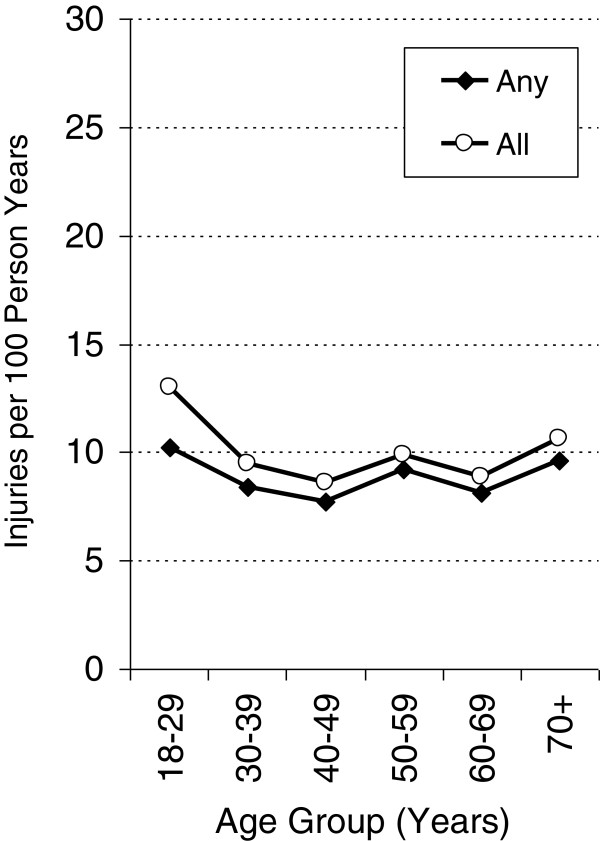
**Age-specific injury rates (per 100 person years) in women.** Any: incidence of any reported accidents during the previous 12 months; All: all reported accidents per 100 person years.

The age-specific incidence pattern of ‘any unintentional injury’ and the total number of injuries per year differed considerably between men and women. Among men, the incidence of ‘any unintentional injury’ steadily decreased from roughly 19.3% among men aged 18–29 years to 4.8% among men aged 70 and over. By contrast, there was little variation in age-based incidence among women. The incidence of the total number of injuries per year was higher than the incidence of ‘any unintentional injury’ particularly among young men (+6.5%) and women (+2.8%) aged 18–29 years, indicating that this age group most often suffered from repeated accident-related injuries in the preceding 12 months (Figures 2 and 3).

**Figure 4:**
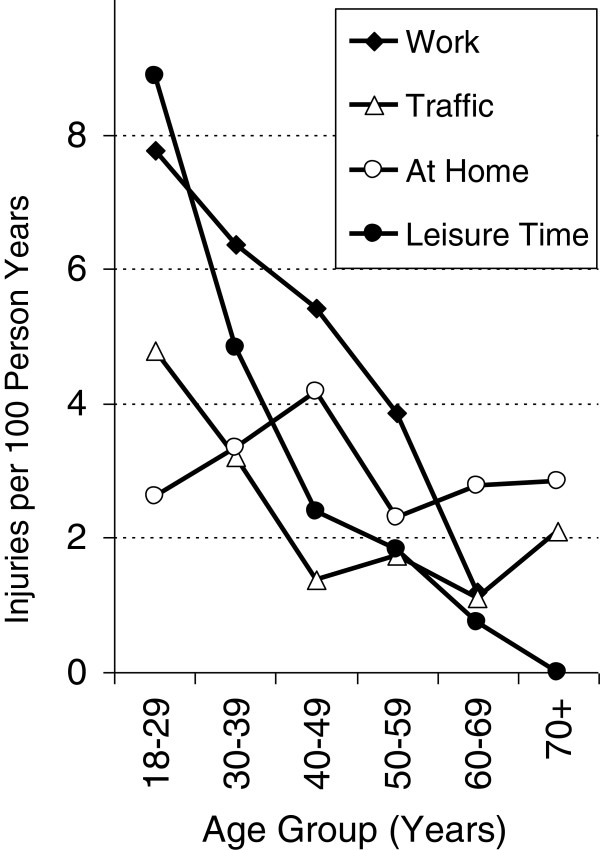
Injury rates by place of accident in relation to age in men.

**Figure 5:**
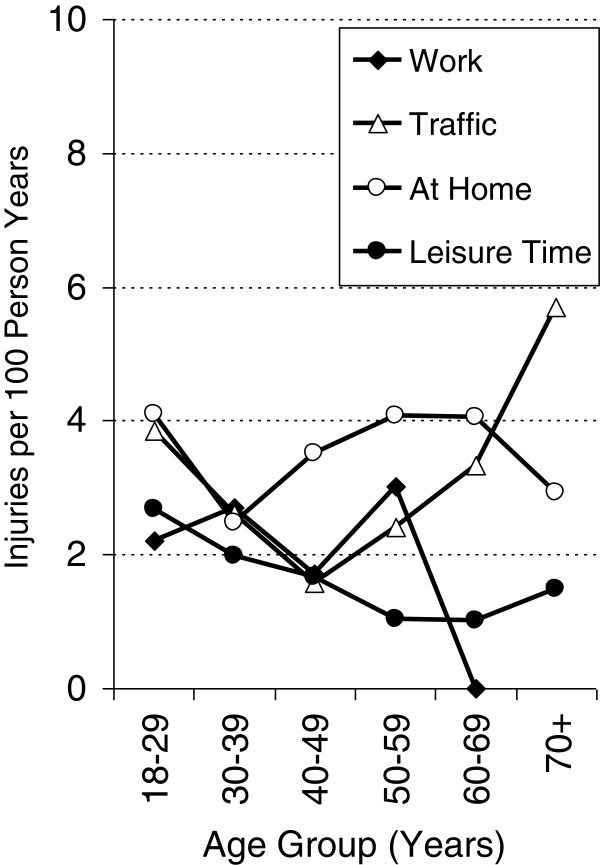
Injury rates by place of accident in relation to age in women.

The age-specific patterns of incidences by place of accident differed among men and women. Among men, the overall steady decrease in injury incidence was mainly driven by the sharp decreases in leisure-time and work-related accidents with increasing age. Interestingly, the incidence of unintentional injuries at home did not substantially vary with age. The incidence of injuries caused by traffic accidents showed a U-shaped age pattern, with the highest incidences occurring among the youngest and oldest age groups. Among women, this U-shape of age-specific traffic incidence was even more prominent. The incidence of work-related injuries and injuries at home barely showed any variation with age among women. Among elderly women (60–69 years), the incidence of work-related injuries was virtually zero. The female incidence of leisure-related injuries steadily decreased up until the age group of 60–69 years; thereafter, it slightly increased (Figures 4 and 5).

The overall injury incidence did not substantially vary with education level. However, the incidences of the different places of accidents differed by educational level. The age-standardised incidence of injuries due to leisure-time accidents was higher among people with a high educational level than those with a low one (men: 3.4% and 1.9%; women: 1.8% and 1.3%, respectively). By contrast, the incidence of work-related injuries was lower among people with a high educational level than those with a low one (men: 2.0% and 6.8%; women: 1.8% and 2.4%, respectively) (Table 3).

**Table 3:** Cumulative incidence of injuries by educational level

	**Men**				**Women**			
	**N**	**CR**	**ASR**	**SE**	**N**	**CR**	**ASR**	**SE**
**Any injury** due to non-fatal accidents (total)								
Low educational level	101	10.3	12.7	1.2	84	8.6	7.7	0.9
Middle educational level	199	13.0	10.6	0.8	200	9.1	9.0	0.7
High educational level	87	9.3	11.6	2.2	54	7.9	7.0	1.0
**All injuries** due to non-fatal accidents (total)								
Low educational level	116	11.8	14.3	1.2	97	9.8	9.7	1.0
Middle educational level	266	17.5	14.1	0.8	230	10.4	10.3	0.7
High educational level	101	10.5	13.0	2.2	58	8.5	7.4	1.1
**Road traffic** accident-related injuries								
Low educational level	17	2.0	2.3	0.5	30	3.9	2.8	0.5
Middle educational level	41	2.6	2.0	0.3	68	3.2	3.2	0.4
High educational level	26	2.8	5.6	2.0	11	1.7	1.6	0.5
**Home** accident-related injuries								
Low educational level	25	2.8	3.7	0.7	33	2.8	3.2	0.7
Middle educational level	60	3.9	3.8	0.5	83	4.0	4.1	0.5
High educational level	22	2.3	2.1	0.5	18	2.8	2.5	0.6
**Leisure** accident-related injuries								
Low educational level	16	1.6	1.9	0.5	14	1.6	1.3	0.4
Middle educational level	65	4.2	3.0	0.4	40	1.5	1.3	0.2
High educational level	33	3.3	3.4	0.7	15	2.1	1.8	0.5
**Workplace**-related injuries*								
Low educational level	43	6.8	6.8	1.0	14	3.0	2.4	0.7
Middle educational level	72	6.9	6.2	1.0	26	2.1	1.8	0.4
High educational level	17	2.5	2.0	0.5	11	2.2	1.8	0.6

N: unweighted number of injuries due to non-fatal accidents; CR: crude rate per 100 person years; ASR: age-standardised rate: weighted age-standardised incidence rate per 100 person years; SE: standard errors of the age-standardised incidence rates calculated using the binomial distribution [12]. Educational status based on the CASMIN educational classification [11].

*Age range restricted to 18–69 years because only 19 people aged 70+ years were still employed.

As the response rate of this survey was low and the survey selection probably favoured respondents with higher educational levels, we standardised the incidence estimates to the educational degree distribution of the census data within the strata of age and gender. These standardised incidences, corrected for potential selection biases, did not markedly differ from the non-standardised incidences. For example, the incidence of injuries caused by leisure-time accidents among subjects aged 20–64 years changed from 4.1% to 3.4% among men and 1.8% to 1.6% among women. The incidence of non-fatal work-related injuries among the working population aged 20–64 years slightly increased from 5.3% to 5.7% among men, but remained unchanged among women.

## Discussion

In this study, we found that about 10.3% of the general German population suffer annually from unintentional non-fatal injuries that require medical treatment. The overall incidence of non-fatal injuries was higher among men than women, and showed different age patterns with regards to gender and place of occurrence. The overall incidence of non-fatal injuries did not substantially vary with the educational levels. However, the places of occurrence (work, home, leisure time and traffic accident) did differ by educational level.

In our survey, we collected a lot of sociodemographic information on the injured individuals, such as age, sex and educational level. Upon analysing these sociodemographic data, we found that work-related injuries had the highest incidence among men. Among both men and women, the incidence of work-related injuries was highest among those with the lowest educational level. Injuries caused by traffic-related accidents most often occurred among young adults (18–29 years) and the elderly (70+ years), and varied in the type of road use (younger: car accidents; elderly: accidents as pedestrians). Altogether, the proportion of pedestrians and cyclists among the people injured in traffic accidents was very high in our study (cyclists: 21.8%, pedestrians 36.6%). Among women, the most frequent place of occurrence was at home. In summing together two important places of accidents, home and leisure time, we observed that they constituted the largest proportion of all reported accident-related injuries (49.5%).

The fact that the injured subjects required medical treatment (as based on self-reports) implies that these accidents were an underestimated burden of disease in Germany. Our survey data may supplement official statistics because we measured injuries (at home, during leisure-time and traffic accidents) that are not necessarily represented in official statistics. We also provided important information on the injury victims, such as age, sex, education level, type of body part injured and hospital admission (the latter two not shown). Such information is necessary to identify high-risk groups and plan specific strategies for injury prevention. Our analyses indicated that young men were especially at high risk of non-fatal injuries. It is also well-documented through cause of death statistics that the risk of fatal injuries in this group is also relatively high [2]. Another high-risk group is vulnerable road users, particularly pedestrians.

Comparing frequency and places of occurrence among published statistics is problematic because categorisations may differ. For example, our estimated one-year incidence of non-fatal injuries (10.3%) was in line with the annual estimates published by the Federal Institute for Occupational Safety and Health [16]. Further agreement was seen in the relative frequency of certain places of accidents. For example, the Institute’s estimation and our results demonstrated that home and leisure injuries (overall) accounted for the most frequent types of injuries (Bundesanstalt für Arbeitsschutz und Arbeitsmedizin 2004: 63.2% [16], Telephone health survey 2004: 49.5%). However, comparing injuries due to leisure-time accidents among the different reported statistics is complicated because the Institute categorises injuries at public places, e.g., when walking or cycling, as leisure-time accidents if they occur during leisure time. On the other hand, in our survey, these injuries were considered to be caused by traffic accidents. In addition, the Federal Institute for Occupational Safety and Health also counted injuries that did not require medical treatment. It is difficult to compare our incidence estimates of traffic accident-related injuries to official road traffic accident statistics by the German Federal Statistical Office because the methodologies in reporting differ. The same is true when trying to compare our incidence estimates of work-related injuries to the workplace accident statistics reported by the statutory accident insurance carriers. For example, our analyses show, like the official traffic accident statistics, a decreasing impact of injuries due to car accidents with increasing age, but a rising number of injured pedestrians in the oldest age groups [17]. However, the percentage of injured pedestrians and cyclists is much higher in our data than in the official road traffic statistics. These accidents may be underreported in the official statistics because they contain only those that are registered by the police.

According to European Union-wide statistics, between 2005 and 2007, about 60 million people - nearly an eighth of EU residents - sought medical treatment for an injury annually (data from the 27 Member States of the European Union) [1]. We obtained a similar incidence, although the EU statistics also included children’s injuries as well as intentional and fatal accidents. Again, the comparison of data on injuries across countries is problematic. Although the collection of data on injuries is considered an important priority in many countries, the actual data collection currently performed in member states varies both in the methodology and in the degree of comprehensiveness of data collection [18]. EU statistics show that home and leisure injuries are the most frequent types of injuries (74.0%) [1]. Our analysis of non-fatal injuries among adults similarly showed that home and leisure together were the most frequent settings for an accident (see above). The difference in the relative frequencies of injuries is due to different inclusion criteria. According to our findings regarding injured pedestrians and cyclists, it is estimated that about half of all hospital-treated injuries are not registered in police statistics [1]. When considering the broader consequences (including economics) of injuries, the European Union is currently seeking to harmonise the methodology of collecting data on this subject across its member countries.

There are several factors that limit our results. First, by nature of the survey used, our results are restricted to non-fatal injuries, as we were only able to interview accident survivors. Moreover, severe injuries that may have resulted in brain damage were underrepresented in our study because the inclusion criteria of the survey required the respondents’ ability to undergo telephone interviews.

Second, we relied completely on self-reports, which are always susceptible to error. As we did not carry out a validation study, we could not report on the sensitivity and specificity of self-reported injuries. We know from other studies that participants remember earlier and/or minor injuries less well, as is also true for injuries in children [19,20]. We asked adults about their own injuries that were treated medically, and so, this did not include minor injuries. Our accident incidences are comparable to those of the Federal Institute for Occupational Safety and Health. Nevertheless, it is possible that a small proportion of injuries were not specified.

Third, the response rate was low. Comparing the distribution of age in our survey with official statistics revealed an underrepresentation of the elderly, especially those over 80 years. However, if we want to calculate age-standardised rates, it is important to take them into consideration because they contribute to the result. Special studies on the frail elderly and those of advanced age will provide a more accurate picture for this group. Comparing the distribution of educational levels in our survey with census data revealed an oversampling of subjects with a higher educational level. Nonetheless, standardisation of the incidence by age- and gender-specific weights based on census data did not substantially change the incidence estimates. Since the telephone survey was conducted in the German language, the number of participants with an immigrant background was below average. There is only little information about their risk of accidents from official statistics. However, we have indications of deviations, for example, more accidents at work in foreigners than in Germans [21]. However, analysing the telephone surveys of the Robert Koch Institute, there was no clear trend as to whether the health status would have improved or not if more immigrants had been included in the survey [22].

## Conclusions

Our investigation emphasised that non-fatal accidents were a frequent health problem in Germany. One in ten adults suffers annually from unintentional non-fatal injuries. Injuries due to accidents are - in Germany and worldwide - associated with high disease burden and high societal costs. For an evaluation of this health problem and of course for injury prevention measures, updated and reliable epidemiological data at the level of the Federal Republic are needed. However, a nationwide comprehensive recording of injuries caused by accidents does not exist. Current available data sources (e.g., police documentation, statistics by statutory accident insurance carriers and statistics related to causes of death) permit only a rough overview of the accident situation in Germany. Data collected in our telephone health survey are an important complement to these sources. Our age- and gender-specific results provide detailed insight into specific patterns of accident-related injuries in Germany. Young men are especially at high risk of injuries. Therefore, the data highlight target groups for injury prevention measures.

At the Robert Koch Institute, a continuous system of health monitoring has been established in recent years [23,24]. It includes repeated nationwide population-based cross-sectional surveys of children and adolescents. Accident-related injuries are an important topic within these surveys. Detailed information on the occurrence of accidents (related to all places of accidents) and its determinants is continuously collected every few years in a module of the survey called "German Health Update" (GEDA). Continuing the comprehensive module “Accident-related injuries” makes current cross-sectional data and information about developments over time available. This includes the possibility of examining accident prevention measures as an important task of health policy.

## Abbreviations

KfV: Kuratorium für Verkehrssicherheit (Austrian Road Safety Board); BAuA: Bundesanstalt für Arbeitsschutz und Arbeitsmedizin(Federal Institute for Occupational Safety and Health); WHO: World Health Organisation; DIMDI: Deutsches Institut für Medizinische Dokumentation und Information(German Institute of Medical Documentation and Information); BMAS: Bundesministerium für Arbeit und Soziales(German Federal Ministry for Labour and Social Affairs); CASMIN: Comparative Analysis of Social Mobility in Industrial Nations; EU: European Union; EU27: 27 EU Member States; ICD-10: International Classification of Diseases and Related Health Problems- 10. Revision

## Competing interests

The authors declare that they have no competing interests.

## Authors’ contributions

ACS participated in the design of the study and drafted the manuscript. AS performed the statistical analysis and helped to draft the manuscript. Both authors read and approved the final manuscript.

## Pre-publication history

The pre-publication history for this paper can be accessed here:

http://www.biomedcentral.com/1471-2458/13/376/prepub
